# Trophic Hierarchies Illuminated via Amino Acid Isotopic Analysis

**DOI:** 10.1371/journal.pone.0076152

**Published:** 2013-09-25

**Authors:** Shawn A. Steffan, Yoshito Chikaraishi, David R. Horton, Naohiko Ohkouchi, Merritt E. Singleton, Eugene Miliczky, David B. Hogg, Vincent P. Jones

**Affiliations:** 1 USDA-ARS, Madison, Wisconsin, United States of America; 2 Department of Entomology, University of Wisconsin, Madison, Wisconsin, United States of America; 3 Institute of Biogeosciences, Japan Agency for Marine-Earth Science and Technology, Yokosuka, Japan; 4 USDA-ARS Yakima Area Research Laboratory, Wapato, Washington, United States of America; 5 Department of Entomology, Tree Fruit Research & Extension Center, Washington State University, Wenatchee, Washington, United States of America; Scottish Association for Marine Science, United Kingdom

## Abstract

Food web ecologists have long sought to characterize the trophic niches of animals using stable isotopic analysis. However, distilling trophic position from isotopic composition has been difficult, largely because of the variability associated with trophic discrimination factors (inter-trophic isotopic fractionation and routing). We circumvented much of this variability using compound-specific isotopic analysis (CSIA). We examined the ^15^N signatures of amino acids extracted from organisms reared in pure culture at four discrete trophic levels, across two model communities. We calculated the degree of enrichment at each trophic level and found there was a consistent trophic discrimination factor (~7.6‰). The constancy of the CSIA-derived discrimination factor permitted unprecedented accuracy in the measurement of animal trophic position. Conversely, trophic position estimates generated via bulk-^15^N analysis significantly underestimated trophic position, particularly among higher-order consumers. We then examined the trophic hierarchy of a free-roaming arthropod community, revealing the highest trophic position (5.07) and longest food chain ever reported using CSIA. High accuracy in trophic position estimation brings trophic function into sharper focus, providing greater resolution to the analysis of food webs.

## Introduction

Stable isotopic analysis has been an indispensable tool of food web ecology [1,2], primarily because the isotopic composition of an organism encodes aspects of its biogeography, physiology, and trophic tendency [3–5]. As matter and energy are transferred among trophic levels, there is discrimination among isotopes at cellular and molecular levels, not only through fractionation but also via isotopic routing [6]. Assimilated isotopes may be effectively stockpiled in certain tissues or certain molecules, while being randomly incorporated within others [7,8]. The inter-trophic shift in a consumer’s isotopic composition relative to its diet has been termed the *trophic discrimination factor* [9] and represents the net effects of enrichment or depletion resulting from fractionation and/or routing [6,10]. While conceptually simple, the predictability of the trophic discrimination factor (Δ = δ_consumer tissue_ −δ_diet_) has remained one of the most vexing, unresolved areas of isotope ecology [1,11,12]. Without a reliable trophic discrimination factor, it is extremely difficult to derive verifiably accurate estimates of the trophic positions of free-ranging animals [13]. Accurate assessment of trophic position is critically important, particularly in an era of climate change, profound biodiversity losses, and “trophic downgrading” [14].

Trophic position estimation via bulk-analysis of the stable nitrogen isotope, ^15^N, has long used +3.4‰ as its trophic discrimination factor (Δ^15^N), given that early studies found this to be the average isotopic increment between trophic levels [3,4,15–17]. Several pioneering aquatic studies were undertaken to characterize the precision of Δ^15^N_bulk_ (bulk-^15^N discrimination factor) and demonstrate its utility in food web studies [16,18]. Early validation of Δ^15^N_bulk_ -derived trophic position estimates, however, was based on gut-content analysis in which the trophic positions of prey were assumed (prey which were, themselves, a mix of omnivores and higher-order predators of unknown trophic positions) [19,20]. Currently, the range of documented Δ^15^N_bulk_ values is known to be quite broad: -2.1 to +9.2‰ [3,16,21–23]. The highly variable Δ^15^N_bulk_ has remained a major problem for trophic ecology, given that the error associated with an imprecise/inaccurate trophic discrimination factor increasingly propagates through the trophic hierarchy of a food web [11].

Compound-specific stable isotope analysis (CSIA) of ^15^N appears to address much of the variability associated with bulk ^15^N-analysis by confining isotopic analyses to select molecules [24–29]. Confining analyses in this way dampens the “noise” generated by the array of nitrogenous compounds that may obscure the more salient “signals” emanating from select compounds. In food web studies, CSIA has generally focused on the suite of essential and non-essential amino acids in autotrophic and heterotrophic biomass [26–29]. Amino acids have proved useful because the ^15^N signatures of certain amino acids enrich very little with each trophic transfer, while others enrich markedly [26,30]. Amino acids enriching very little tend to mirror the ^15^N signature of the resources at the base of the food web. These compounds have been termed *source amino acids*, while those enriching substantially with each trophic transfer have been termed *trophic amino acids* [27,28]. Source amino acids (e.g., phenylalanine), experience only slight enrichment because during most metabolic processes, these amino acids rarely form or cleave carbon-nitrogen bonds [31]. Conversely, trophic amino acids (e.g., glutamic acid) tend to experience higher ^15^N-enrichment because their carbon-nitrogen bonds are commonly cleaved during metabolic transamination, allowing greater opportunities for isotopic discrimination [31]. Inter-trophic enrichment of phenylalanine, in particular, has been observed to be quite small (0.4 ± 0.5‰) while glutamic acid has been relatively high (8.0 ± 1.2‰) [29,32,33]. Not surprisingly, the respective ^15^N signatures of phenylalanine and glutamic acid follow divergent enrichment trajectories as a consumer feeds higher in its trophic hierarchy [29]. It is the predictability of this divergence that has made these two amino acids ideal candidates for determining trophic position.

To-date, the CSIA approach has been used to estimate the trophic positions of consumer species in marine, freshwater, and terrestrial ecosystems [27,29,33–36]. By measuring the ^15^N signatures of source and trophic amino acids extracted from the homogenized biomass of an animal, the disparity between the two signatures can be calculated. To the extent that there exists a valid trophic discrimination factor, the trophic position of the animal can be accurately determined [29,33]. However, as with the early bulk-^15^N studies, it is necessary to validate the accuracy of CSIA-based trophic position estimates, ideally with methods independent of isotopic analysis. Early CSIA studies involving consumers in pure culture and fed known diets suggested that Δ^15^N_glu-phe_ averaged approximately +7.6‰ [26,29,31,32], although these studies involved relatively few specimens (N = 12), most of which were marine herbivores (N = 9). The Δ^15^N_glu-phe_ estimate, therefore, was derived from limited empirical data and was largely confined to a single trophic group.

To show that the Δ^15^N_glu-phe_ was broadly stable among higher-order consumers, particularly predators of predators, we created four discrete trophic groups and then used CSIA to determine the degree of isotopic enrichment between trophic levels. Our data from two separate controlled-feeding trials, representing two distinct communities, address explicitly whether there is a consistent, non-scaling trophic discrimination factor, and whether this factor is centered around +7.6‰. Next, employing an ecosystem-specific formula for trophic position estimation [33], we tested the accuracy of the CSIA approach using only organisms of known trophic position. Finally, we brought this approach to the field and examined a trophic hierarchy composed of wild, free-roaming arthropods. Our work provides the first evidence of a trophic discrimination factor that does not scale with trophic level, nor does it appear to change among ecosystem types. Using this discrimination factor, the accuracy and precision of all trophic position estimates were extraordinarily high, providing greater resolution to assessments of trophic function among free-roaming fauna.

## Materials and Methods

### Controlled-feeding trials

Two controlled-feeding trials were conducted, one involving an isotopically heterogeneous basal resource (bean plants) and the other, an isotopically homogeneous resource (homogenized oats and cranberries). In the first trial, bean plants (*Phaseolus vulgaris* L.) were propagated in a greenhouse using sterilized soil from an old-field site at the USDA-ARS Yakima Area Research Laboratory (Wapato, WA). A pure culture of pea aphids (*Acyrthosiphon pisum* Harris) was established on the bean plants. Green lacewing eggs (collected from an apple orchard in Quincy, Washington) were allowed to hatch, identified ( *Chrysopa* *nigricornis* Burmeister), individually separated into microcosms, and fed the pea aphids. These lacewings represented “strict predators” (= trophic level 3.0) given their strict diet of herbivores. A subset of the newly hatched lacewings destined to be trophic level 4.0 (TL4) was separated and fed only the TL3 larvae. All consumed larvae had been frozen (to ensure that the consumer did not become the “meal”) and then thawed before provisioning. All lacewing larvae were fed until they pupated. Plant, aphid, and adult lacewing specimens were dried, weighed, and packed in tin capsules for bulk ^15^N-analysis (4-7 samples of each trophic group were prepared, depending on available biomass). Aliquots from each specimen were placed in separate vials for amino acid extractions via the Chikaraishi method (see *Aminoacid* *extraction and isotopic analysis* below).

In the second trial, a homogeneous blend of oats (*Avena sativa* L.) and cranberries ( *Vaccinium* *macrocarpon* Ait.) was created. The insect diet was confined to these two ingredients because the herbivore species in this trial, fall armyworm ( *Spodoptera* *frugiperda* Smith), is a pest of grain crops and cranberries. Standard insect diets could not be used because we needed to ensure that all elements of the food chain were known. Approximately 2.9 liters of boiled oats (oatmeal) was made and then 360 mL of raw cranberries were added before completely homogenizing the two ingredients in a blender. To each cell of twelve 128-cell plastic trays, ~2 ml of the cranberry-oatmeal blend was added. The diet was allowed to cool and then desiccate for 18-20 h (drying at the surface of the cranberry-oatmeal blend was important to eliminate surface tackiness, which was lethal for small caterpillars). Ten samples of diet were isolated and dried for subsequent isotopic analyses. Fall armyworm eggs that had been purchased (3,000 eggs, from Bio-Serv, Inc.) were incubated until eclosion. Neonate larvae were then placed into each cell of the diet trays (two larvae per cell) and incubated at 30°C. As larvae grew and molted, individuals were removed and frozen, to serve as future prey for the carnivore groups. Diet cells with a single larva remaining were not included (to eliminate the possibility of cannibalism within the herbivore group). Approximately 600 2^nd^-instar armyworm larvae were banked (frozen) after 3 days of feeding; on day-4, 800 3^rd^-instar larvae were banked; on day-5, 250 4^th^-instars were banked, and on day-6, 110 5^th^-instars were banked. Ten 5^th^-instar larvae were set aside for isotopic analysis. With adequate numbers of herbivores banked, eggs of the carnivore group, green lacewings ( *Chrysoperlarufilabris* Burmeister), were purchased (Rincón-Vitova Insectaries, Inc., Ventura, CA). Lacewing eggs (1,000) were incubated at ~25°C until eclosion; as the larvae began to emerge, each larva was placed in a microcosm and fed a 2^nd^-instar armyworm larva. These lacewings represented trophic level 3 (TL3). Five hundred 1^st^-instar lacewings were fed a 1^st^-instar armyworm larva. As the lacewings grew and molted to subsequent instars over the course of ~10 days, the size of their prey was increased accordingly. A subset of the newly hatched lacewings destined to be trophic level 4.0 (TL4) was separated and fed only frozen TL3 larvae. At each of the four trophic levels in this trial, a minimum of eight samples was prepared for both bulk-^15^N and CSIA analysis. Bulk ^15^N samples were submitted to the Washington State University Stable Isotope Core Lab (http://www.isotopes.wsu.edu/services.html) for analysis. Data are reported as the ‰ departure from a standard (atmospheric N_2_): [(*R*_sample_/*R*_standard_) -1)] × 1,000.

### Orchard food web

Plant and arthropod specimens were collected from mature apple orchards in the Pacific Northwest (Wenatchee and Quincy, WA), USA. Private grower-collaborators in these regions were active participants in our work, and permitted routine sampling of invertebrates in their orchards. Samples of apple leaves ( *Malus* *domestica* L.), apple aphids (*Aphis pomi* DeGeer), hover flies ( *Eupeodes* spp.), parasitoids (*Bothriothorax* near *rotundiformis*), and hyperparasitoids ( *Pachyneuronalbutius* ) were collected, curated, and identified in 2009, 2010, and 2012. Identifications of the parasitoids were accomplished by Robert Zuparko (California Academy of Sciences, San Francisco, CA); the hyperparasitoids were identified by Steven Heydon (University of California-Davis, Davis, CA). *Pachyneuronalbutius* , a pteromalid wasp (Hymenoptera), is a parasitoid of *Bothriothorax* sp., an encyrtid wasp (Hymenoptera), which commonly parasitizes hover fly puparia [37]. While hover flies in apple orchards are generally pollen- and nectar-feeders as adults, their larvae are voracious predators, specializing on abundant aphid populations [38]. Apple aphids are small sap-feeding herbivores, very common to apple orchards. All specimens were analyzed using both the bulk and CSIA methods. *δ* ^13^C values were also determined to verify that all specimens were part of C_3_ plant food webs.

### Amino-acid extraction and stable isotope analysis

The nitrogen isotopic composition (*δ* ^15^N) of glutamic acid and phenylalanine were determined by gas chromatograph/combustion/isotope ratio mass spectrometer (GC/C/IRMS) after HCl hydrolysis and *N*-pivaloyl/isopropyl (Pv/iPr) derivatization, according to established procedures (see “Preparation and ^15^N/^14^N analysis of amino acids” at http://www.jamstec.go.jp/biogeos/j/elhrp/biogeochem/download_e.html). In brief, samples were hydrolyzed using 12 M HCl at 100°C. The hydrolysate was washed with *n*-hexane/dichloromethane (3/2, v/v) to remove hydrophobic constituents. Then, derivatizations were performed sequentially with thionyl chloride/2-propanol (1/4, v/v) and pivaloyl chloride/dichloromethane (1/4, v/v). The Pv/iPr derivatives were extracted with *n*-hexane/dichloromethane (3/2, v/v). The nitrogen isotopic compositions were determined by GC/C/IRMS using an Agilent Technologies 6890N GC coupled to a Thermo, Fisher Scientific Delta ^plus^XP IRMS with a GC-C/TC III interface, with an analytical error in δ^15^N being less than 0.5‰.

### Calculation of the trophic discrimination factor and trophic position

The trophic discrimination factor (Δ^15^N_glu−phe_) was calculated as the difference in enrichment between a consumer and its diet, with respect to glutamic acid (glu) and phenylalanine (phe):

Δ^15^N_glu−phe_ = (*δ* ^15^N_consumer_ −*δ* ^15^N_diet mean_)_glu_ - (*δ* ^15^N_consumer_ −*δ* ^15^N_diet mean_)_phe_ (1)

where *δ* ^15^N_phe_ represented the isotopic signature of phenylalanine, and *δ* ^15^N_glu_ represented that of glutamic acid. As with *δ* ^15^N notation, the unit of measure for Δ^15^N_glu−phe_ is ‰. In Equation (1), the expression “(*δ* ^15^N_consumer_ −*δ* ^15^N_diet mean_)_glu_” denotes the difference in *δ* ^15^N_glu_ between the consumer and its diet, and “(*δ* ^15^N_consumer_ −*δ* ^15^N_diet mean_)_phe_” denotes the difference in *δ* ^15^N_phe_ between the consumer and its diet. Since the actual food ingested and assimilated by any given consumer cannot be readily assayed for its ^15^N signature, a mean is derived from this diet (*δ* ^15^N_diet mean_). By quantifying the enrichment of phenylalanine and then subtracting this value from the enrichment of glutamic acid, we effectively isolate the enrichment of ^15^N due to the inter-trophic transfer of N [29,33].

In previous studies [29,32], the trophic position (TP_glu-phe_) of a given specimen was calculated from the observed *δ* ^15^N values of glutamic acid and phenylalanine using the following equation:

TP_glu-phe_ = [(*δ* ^15^N_glu_ -*δ* ^15^N_phe_ + β) / Δ^15^N_glu−phe_] + 1 (2)

where *δ* ^15^N_glu_ represents the isotopic signature of glutamic acid, and *δ* ^15^N_phe_ represents the signature of phenylalanine. The parameter, β, in Equation (2) represents the disparity between phenylalanine and glutamic acid signatures within the basal resource of any given food web, and is calculated as β = (*δ* ^15^N_phe_ −*δ* ^15^N_glu_)_basal resource_ [29]. The β term varies substantially among ecosystem types (i.e., marine, freshwater, terrestrial C_3_ plant systems, and C4-plant systems), and can vary within ecosystem types: in terrestrial food webs (C_3_ plant species), β has been reported to average +8.4 ± 1.6‰ [33]. In the present study, the basal resources were known, so a mean value of β could be determined for each particular food web.

 Trophic position estimates based on bulk ^15^N-analysis were calculated as

TP_bulk_ = [(*δ* ^15^N_consumer_ -*δ* ^15^N_basal resource mean_) / 3.4‰] + 1 (3)

based on previous studies where the trophic discrimination factor was assumed to be +3.4‰ [4,16,33]. In our controlled-feeding study, empirical estimates of the trophic discrimination factors for bulk ^15^N-analyses, Δ^15^N_bulk_, were calculated as Δ^15^N_bulk_ = *δ* ^15^N_consumer_ -δ^15^N_diet average_.

### Statistical analyses

Variability in the observed Δ^15^N_glu−phe_ and Δ^15^N_bulk_ values were assessed within and across trophic levels via replicated regression analysis [39]. Two-way ANOVA was used to examine how the two different discrimination factors, Δ^15^N_glu−phe_ and Δ^15^N_bulk_, varied with Trophic Level and Trial; planned contrasts between trophic levels were conducted using pair-wise tests (Fisher LSD). Linear regression analysis was used to provide evidence of any broader trend across trophic levels. Univariate analysis was then used to determine whether the observed Δ^15^N_glu−phe_ diverged from the established +7.6‰ value. Replicated regression analysis was used to assess Δ^15^N_bulk_ variability over trophic levels and trials; univariate tests were run to determine whether the observed Δ^15^N_bulk_ diverged significantly from the established +3.4‰. The accuracy of trophic position estimates was assessed using paired *t*-tests, wherein the known TP of a given organism was compared to the observed TP for that organism. Non-parametric tests (Mann-Whitney rank sum tests) were used where data did not conform to assumptions of normality or homogeneous variances.

## Results

### Trophic discrimination factors

**Figure 1:**
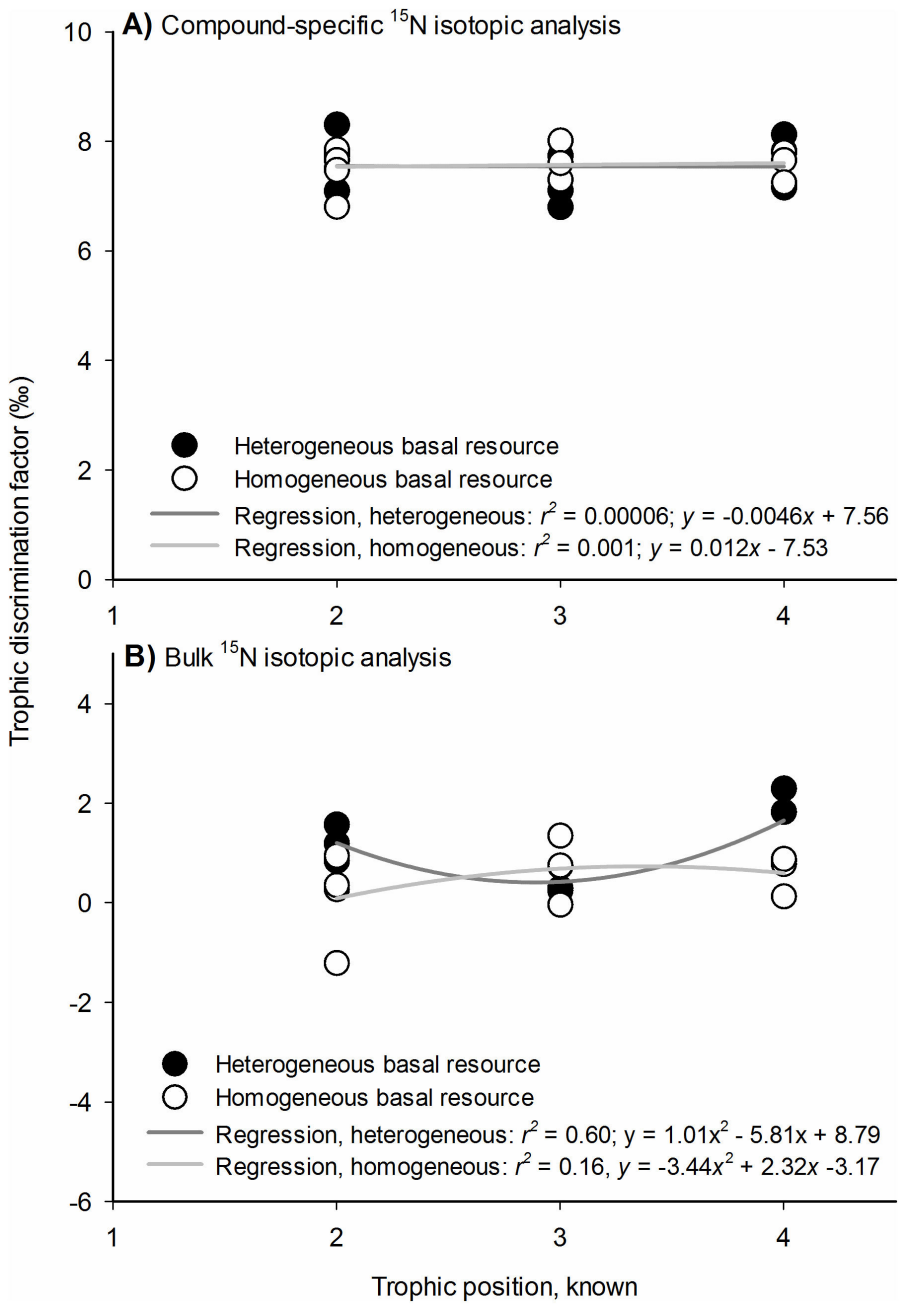
Trophic discrimination factors. Linear regression analysis of trophic discrimination factors (Δ^15^N) deriving from A) CSIA analysis, and B) bulk ^15^N analysis. Each point represents the trophic discrimination factor of an individual organism. Discrimination factors depicted at trophic levels 2, 3, and 4 represent the isotopic shifts from levels 1 to 2, 2 to 3, and 3 to 4, respectively.

Using CSIA to measure the ^15^N signatures of glutamic acid and phenylalanine, the mean trophic discrimination factor (Δ^15^N_glu-phe_) in our controlled feeding study was +7.56 ± 0.089‰ (± SE), with a median value of +7.66‰. Mean Δ^15^N_glu-phe_ was consistent within and among all trophic levels and trials (Trophic Level × Trial: *F*_*2,15*_ = 0.86, *P* = 0.44; Trophic Level main effect: *F*_*2,15*_ = 0.61, *P* = 0.56; Trial main effect: *F*_*1,15*_ = 0.008, *P* = 0.93). From trophic level 1.0 to 2.0, Δ^15^N_glu-phe_ was +7.61 ± 0.19‰. From level 2.0 to 3.0, Δ^15^N_glu-phe_ was +7.43 ± 0.14‰, and for level 3.0 to 4.0, it was +7.62 ± 0.13‰. Linear regression analyses further support the constancy of Δ^15^N_glu-phe_ over the range of trophic levels investigated (Figure 1A); in either of the controlled-feeding trials (see *Materials and Methods*), there was no significant evidence to suggest that the slope terms were non-zero (heterogeneous basal resource, slope term: *P* = 0.984, *y*-intercept: *P* < 0.001, *R* ^2^ = 0.00006; homogeneous resource, slope term: *P* = 0.92; *y*-intercept: *P* < 0.001, *R* ^2^ = 0.001). Across both trials, mean Δ^15^N_glu-phe_ was not divergent from the established CSIA discrimination factor, +7.6‰ (univariate *t* = -0.503, df = 20, *P* = 0.62).

Using bulk analysis of ^15^N signatures in the controlled-feeding study, the degree of trophic enrichment (Δ^15^N_bulk_) changed significantly among trophic levels and trials (Trophic Level × Trial: *F*_*2,13*_ = 5.61, *P* = 0.018). Within the heterogeneous resource trial, mean Δ^15^N_bulk_ at trophic levels 2.0, 3.0, and 4.0 were, respectively, +1.20 ± 0.34‰, +0.42 ± 0.34‰, and +1.65 ± 0.34‰ (Table S1). At trophic level 4.0, mean Δ^15^N_bulk_ represented a significant increase from that registered at trophic level 3.0 (pairwise comparison, Fisher LSD: *P* = 0.024). Regression analysis indicated there was a significant parabolic relationship between Δ_bulk_ and trophic level in the heterogeneous food web (regression fit: *F*_*2, 15*_ = 9.75, *P* = 0.0026, *R* ^2^ = 0.60; Figure 1B), and that a simple linear model was non-predictive (slope term: *P* = 0.140, *y*-intercept: *P* = 0.713; *R* ^2^ = 0.010). Within the homogeneous diet trial, mean Δ^15^N_bulk_ at trophic levels 2.0, 3.0, and 4.0 were, respectively, +0.38 ± 0.29‰, +0.96 ± 0.34‰, and -0.067 ± 0.34‰. There was marginal evidence that Δ^15^N_bulk_ differed significantly between trophic levels 3.0 and 4.0 (pairwise comparison, Fisher LSD: *P* = 0.052), but not for either of the other two trophic levels (pairwise comparison of 2.0 vs. 3.0: *P* = 0.22; pairwise comparison of 2.0 vs. 4.0: *P* = 0.33). Mean Δ^15^N_bulk_ across all trophic positions and both trials was +0.74 ± 0.18‰, a significant departure from the conventional +3.4‰ discrimination factor (*t* = -15.15, df = 18, *P* < 0.001).

### Trophic position estimates

**Figure 2:**
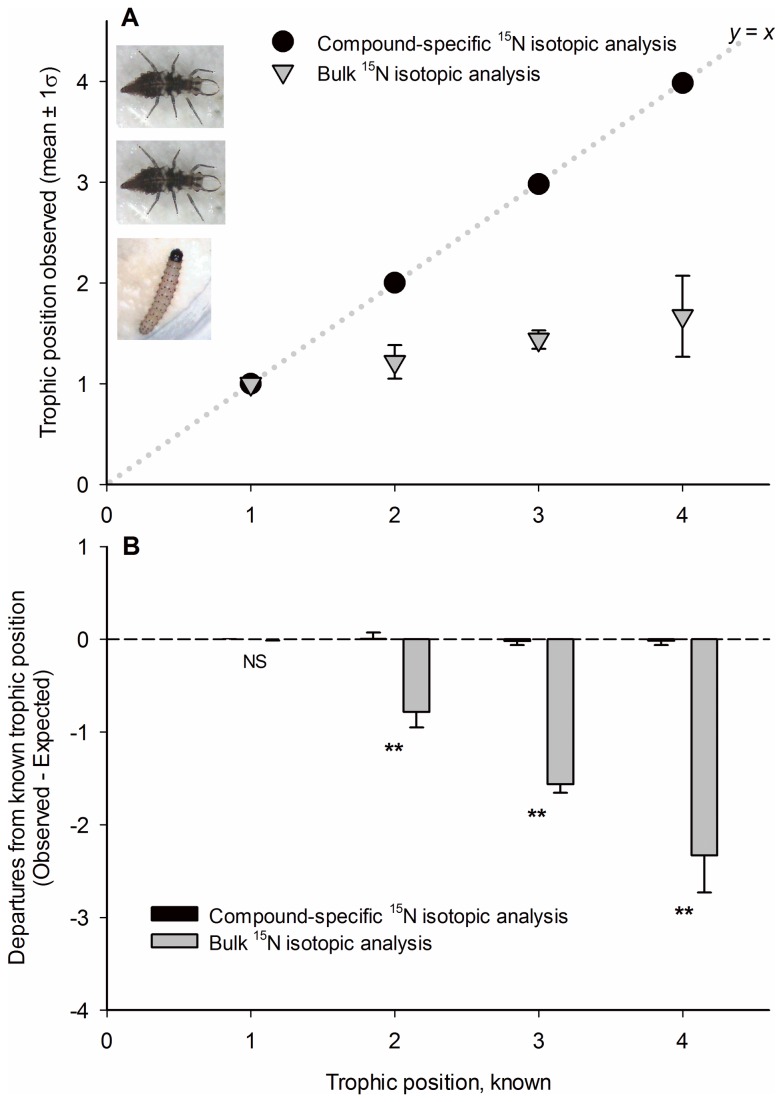
Trophic position estimates, controlled-feeding trials. Trophic position estimates (mean ± 1σ) from the controlled-feeding trials. A) Plots of observed trophic positions vs. their corresponding known trophic positions. Black circles indicate TP_glu-phe_ estimates, and gray triangles indicate TP_bulk_ estimates. The dotted line (*y* = *x*) represents perfect agreement between observed and known trophic positions. B) Degree of departure between the observed and known trophic positions (mean ± 1σ).

Trophic position estimates generated using compound-specific isotopic analysis (with Δ^15^N_glu-phe_ = 7.6‰) were exceedingly accurate (Figure 2A-B). On average, the TP_glu-phe_ estimates diverged from their respective TP_known_ values by 0.0092 ± 0.0085, an insignificant departure (*t* = 1.08, df = 27, *P* = 0.289). Accuracy was significantly improved by using the community-specific β value (Mann-Whitney rank sum test: *T* = 1,186.5, *P* < 0.001; Table S1, S2), as opposed to the standard β value (+8.4‰) established for terrestrial C_3_ plant food webs [33].

TP_bulk_ estimates diverged from the TP_known_ values by 1.11 ± 0.18, a highly significant departure (*t* = 6.24, df = 25, *P* < 0.001). Within the basal trophic group (trophic level 1.0), the CSIA and bulk-analysis methods were similarly accurate (*t* < 0.001, df = 12, *P* = 1.0; Figure 2B). Within the other three trophic groups (trophic levels 2.0, 3.0, and 4.0), the TP_glu-phe_ estimates were significantly more accurate than those of TP_bulk_ (trophic level 2.0: Mann-Whitney rank sum test, *T* = 77.0, P < 0.001; trophic level 3.0: *t* = 39.67, df = 11, *P* < 0.001; trophic level 4.0: *T* = 21.0, *P* = 0.001).

**Figure 3:**
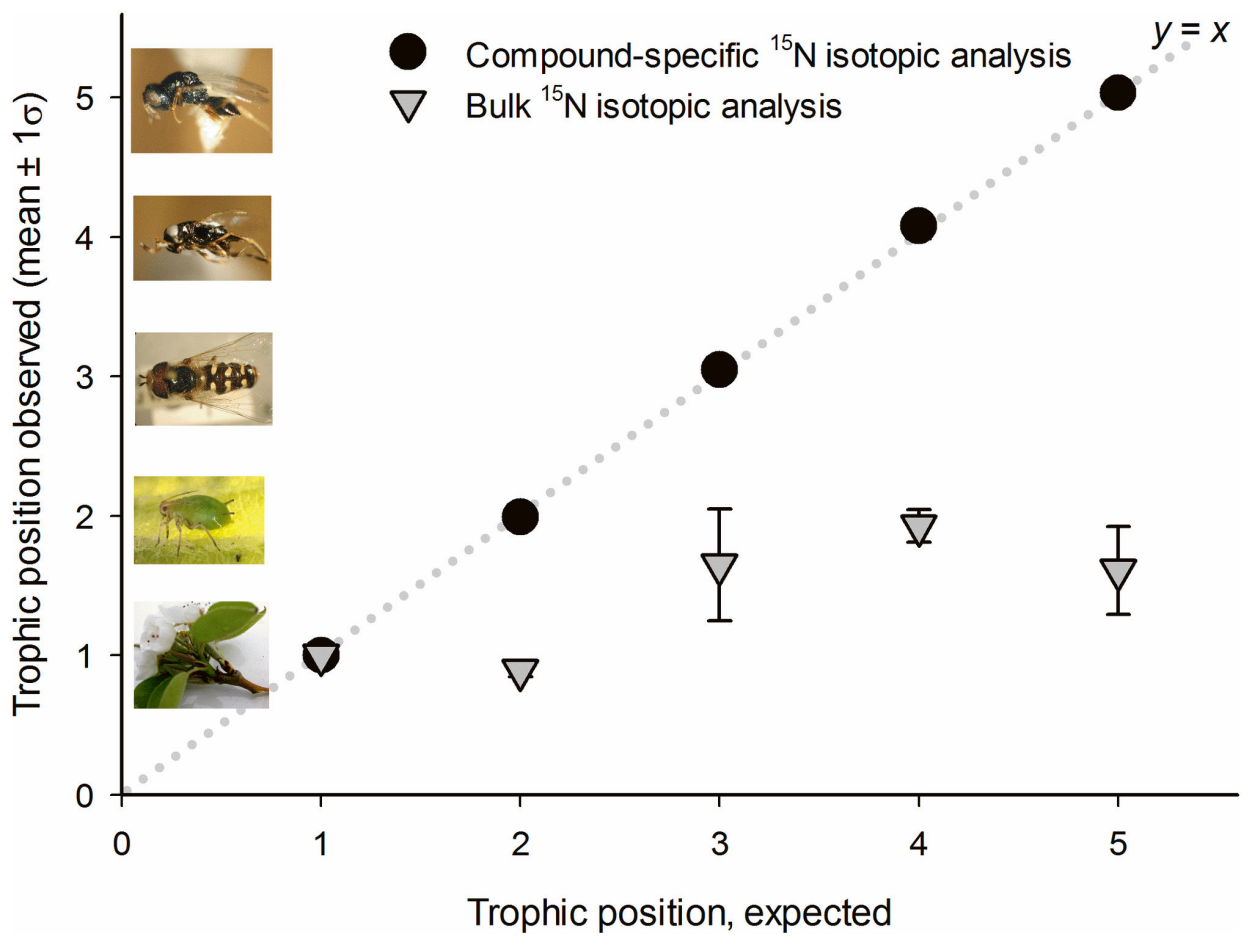
Trophic position estimates, apple orchard. Trophic position estimates (mean ± 1σ) from an apple orchard food chain (pictured): apple leaves, apple aphid, hover fly, parasitoid, and hyperparasitoid. Observed trophic positions are plotted against expected trophic positions. Black circles and gray triangles respectively indicate TP_glu-phe_ and TP_bulk_ estimates. The dotted line (*y* = *x*) represents perfect agreement between observed and expected trophic positions.

The wild food-chain produced similar results (Fig. 3; Table S2). Five species were analyzed, each representing a distinct trophic group with narrow, specialized feeding habits. Using CSIA, the mean difference between observed and expected trophic levels was -0.031 (± 0.014) which, though relatively small, was a significant departure from the expected trophic positions of the specimens (*t* = -2.22, df = 19, *P* = 0.039). Here, the accuracy of the CSIA approach was exceeded by its precision. Using bulk-analyses, the mean difference between observed and expected trophic levels was 1.58 (± 0.26), a significant departure from the expected trophic positions of the specimens (*t* = 6.06, df = 19, *P* < 0.001).

## Discussion

The trophic position of an animal has remained remarkably difficult to measure accurately [1,21]. Using compound-specific isotopic analysis, we provide the first evidence that ^15^N enriches consistently among trophic levels 2.0, 3.0, and 4.0. This range of trophic activity represents the majority of global fauna (i.e., all herbivores, omnivores, strict predators, and most tertiary predators). Not only was this trophic discrimination factor, Δ^15^N_glu-phe_, constant across the range of trophic levels in our trials, but it was also centered closely around +7.6‰, validating the discrimination factor previously reported among marine and aquatic herbivores [29,32]. The degree of consistency across multiple trophic levels, as well as across major ecosystem types, suggests that Δ^15^N_glu-phe_ may be relatively portable among ecosystems.

Having shown that Δ^15^N_glu-phe_ was non-scaling across a range of trophic levels (Figure 1), we tested the accuracy of an ecosystem-specific formula for trophic position estimation (terrestrial C_3_ plant formula, per Chikaraishi et al. 2011). We assembled two communities, each comprised of four discrete trophic groups, thereby creating organisms of known trophic position. These organisms ranged from autotrophs (plant biomass) to apex predators. By coupling CSIA with the terrestrial C_3_ plant formula, we generated trophic position estimates that diverged from their respective, known trophic positions by < 1% (Figure 2; Table S1). These findings suggest that the trophic tendency of an animal can be measured with high accuracy when ^15^N analyses are confined to glutamic acid and phenylalanine. Limiting the analyses to these two amino acids effectively screens what would otherwise be a very heterogeneous mix of ^15^N signatures.

We then brought the CSIA method to bear upon a community of arthropods representing five trophic groups in a terrestrial food web. Again, CSIA coupled with the Chikaraishi C_3_ plant formula produced trophic position estimates that were close to the expected trophic position of each taxon in the food web (Figure 3). Individuals of the wasp species, *Pachyneuronalbutius* , repeatedly registered a trophic position of 5.07 in our study (Table S2). This wasp represents the highest trophic position ever reported using CSIA. *P* *. albutius* is a specialist on the parasitoids (*Bothriothorax*) that attack hover fly puparia in Washington apple orchards. Since the hover flies are, themselves, predators of aphids (thus, expected trophic level ~3.0), the *Bothriothorax* wasps that attack them should be near 4.0; *P* *. albutius* wasps were expected to register near 5.0. The confirmation of this high trophic position indicates that a five-level food chain is not only possible but also fairly common among arthropods, given the diversity of hyperparasitoids known to attack predator species in terrestrial systems [37]. The resulting trophic hierarchy in the apple orchard represents the longest food chain ever reported where the trophic positions of consumers were empirically measured with high precision and accuracy.

Within this orchard food web, our trophic position estimates diverged from the expected trophic levels by just -0.031, which represented a relatively small 3.1% departure. Across the three communities we examined, accuracy was significantly improved by using the community-specific β value in the trophic position calculation (Table S1, S2). While the standard β value of +8.4‰ [33] allowed for very accurate trophic position estimates, using a community-specific β better addressed the issue of background variability (Table S1).

The bulk-^15^N trophic position estimates in our controlled-feeding study were notably inaccurate, diverging by 1.11 trophic levels, on average. This degree of inaccuracy would dramatically alter the perceived trophic niche of a species. For example, when the trophic positions of the organisms in our controlled-feeding studies were assayed via bulk-^15^N analysis, the carnivores (i.e., trophic levels 3.0 and 4.0) were indistinguishable from the herbivores (Figure 2). The general inaccuracy in trophic position estimates using bulk-^15^N methods derives from idiosyncratic background signatures and the highly variable Δ^15^N_bulk_ [1]. Background heterogeneity can be accommodated with careful experiments and statistical rigor [15,16,18,40], but a consistent trophic discrimination factor has remained a critical, missing element. The primary consequence (and irony) of the widely varying Δ^15^N_bulk_ is that the trophic position estimate for any given specimen may be highly inaccurate, even though the ^15^N ratio of the specimen has been measured accurately. System-specific and consumer guild-specific Δ^15^N_bulk_ values have been generated to address this problem (e.g., see Vander Zanden et al. 2001), but in most cases, isolating the trophic discrimination factor for each ecosystem type and/or trophic group for wide-ranging carnivores is prohibitively difficult; hence, a general Δ^15^N_bulk_ value from the published literature is often relied upon for trophic position estimation, regardless of whether the species, diet types, or tissues correspond to the focal organism [41].

The historical lack of a means to accurately characterize the trophic niches of animals has forced food web ecologists to consolidate species into broad trophic subsets, such as “carnivore” and “omnivore;” clearly, there is a need for greater resolution in the measurement of trophic attributes [42,43]. Relegating species to coarse-grain classifications effectively overlooks vertical diversity and lumps together omnivore and carnivore groups that may have countervailing impacts on primary production [14,44]. As food chains lengthen it becomes increasingly important to understand how the loss of any single trophic group will impact the ecosystem [14]. Our data reveal how phenylalanine and glutamic acid signatures enrich predictably across a wide range of trophic levels. Phenylalanine signatures changed very little while those of glutamic acid enriched significantly with each trophic transfer. Equally important was our finding that background heterogeneity in ^15^N was captured in the phenylalanine signature of each specimen. The phenylalanine ^15^N signature effectively provided a steady “platform” on which glutamic acid’s ^15^N signature could reveal enrichment due solely to trophic mechanisms. Thus, it was the combination of the stable discrimination factor and the baseline information of phenylalanine that facilitated the accurate trophic position estimates in our experiments. The capacity of CSIA to accurately characterize the lifetime trophic tendency of a free-roaming animal will allow researchers to determine the degree to which consumer species indulge in omnivory, as well as the degree to which carnivores attack fellow carnivores. Importantly, formalin and other common preservatives do not adversely affect the accuracy of CSIA-based trophic position estimates [45]. This means that old specimens can be exhumed from museum drawers and analyzed for their trophic positions. CSIA, therefore, not only provides highly resolved images of functional diversity within contemporary food webs, but also permits the examination of food webs that have long since perished.

## Supporting Information

Table S1 **Measurements and calculations from the two controlled-feeding trials.**
(DOCX)

Table S2 **Measurements and calculations from the orchard food web.**
(DOCX)
