# Supplementary material for: Trophic Hierarchies Illuminated via Amino Acid Isotopic Analysis
Source: PLoS One. 2013 Sep 25;8(9):e76152. doi: 10.1371/journal.pone.0076152 (PMC3783375; doi:10.1371/journal.pone.0076152)
Supplement: Table S1 — Measurements and calculations from the two controlled-feeding trials. (DOCX) [file pone.0076152.s001.docx]

**Table S1.** Observations and calculations from two controlled-feeding trials.

| **Sample**  **type** | **TP_k_** | ***δ*^15^N_bulk_** | **Δ^15^N_bulk_** | **TP_bulk_** | ***δ*^15^N_glu_** | ***δ*^15^N_phe_** | ***β*** | **Δ^15^N_glu-phe_** | **TP_glu-phe_** | **TP^‡^_glu-phe_** |
| --- | --- | --- | --- | --- | --- | --- | --- | --- | --- | --- |
| *Trial 1 (heterogeneous basal resource)* | | | | | | | | | |  |
| Bean plant | 1.0 | 1.57 |  | 1.01 | 2.79 | 10.06 | 7.26 |  | 1.15 | 1.00 |
|  | 1.0 | 1.48 |  | 0.98 | 2.97 | 10.17 | 7.20 |  | 1.16 | 1.00 |
|  | 1.0 | 1.59 |  | 1.01 | 0.67 | 7.92 | 7.25 |  | 1.15 | 1.00 |
| Pea aphids | 2.0 | 2.39 | 0.84 | 1.25 | 6.81 | 6.95 |  | 7.10 | 2.09 | 1.93 |
|  | 2.0 | 2.74 | 1.19 | 1.35 | 5.96 | 5.46 |  | 7.74 | 2.17 | 2.02 |
|  | 2.0 | 3.12 | 1.57 | 1.46 | 7.49 | 6.43 |  | 8.31 | 2.25 | 2.09 |
| Lace-wing | 3.0 | 3.04 | 0.29 | 1.44 | 8.43 | 0.85 |  | 7.11 | 3.10 | 2.95 |
|  | 3.0 | 3.48 | 0.73 | 1.57 | 8.99 | 0.78 |  | 7.74 | 3.19 | 3.03 |
|  | 3.0 | 3.00 | 0.25 | 1.43 | 3.63 | -3.65 |  | 6.80 | 3.06 | 2.91 |
| Lace-wing | 4.0 | 4.01 | 0.84 | 1.72 | 11.73 | -3.79 |  | 7.83 | 4.15 | 3.99 |
|  | 4.0 | 5.00 | 1.83 | 2.02 | 11.63 | -4.19 |  | 8.13 | 4.19 | 4.03 |
|  | 4.0 | 5.47 | 2.30 | 2.15 | 12.56 | -2.29 |  | 7.16 | 4.06 | 3.91 |
| *Trial 2 (homogeneous basal resource)* | | | | | | | | | |  |
| Cran-berry oatmeal | 1.0 | 3.80 |  | 0.98 | 1.46 | 8.65 | 7.19 |  | 1.16 | 1.00 |
|  | 1.0 | 3.84 |  | 1.00 | 1.09 | 8.20 | 7.10 |  | 1.17 | 1.01 |
|  | 1.0 | 3.88 |  | 1.01 | 1.11 | 8.33 | 7.22 |  | 1.16 | 0.99 |
|  | 1.0 | 3.90 |  | 1.01 | 1.33 | 8.52 | 7.20 |  | 1.16 | 1.00 |
| Fall army-worm | 2.0 | 4.80 | 0.95 | 1.28 | 9.01 | 8.68 |  | 7.51 | 2.15 | 1.99 |
|  | 2.0 | 4.13 | 0.28 | 1.08 | 9.38 | 8.74 |  | 7.82 | 2.19 | 2.03 |
|  | 2.0 | 4.21 | 0.36 | 1.10 | 8.59 | 8.96 |  | 6.81 | 2.06 | 1.90 |
|  | 2.0 | 3.82 | -0.04 | 0.99 | 9.36 | 8.52 |  | 8.02 | 2.22 | 2.05 |
| Lace-wing | 3.0 | 5.59 | 1.35 | 1.51 | 17.04 | 9.38 |  | 7.30 | 3.11 | 2.95 |
|  | 3.0 | 4.99 | 0.75 | 1.33 | 17.23 | 9.26 |  | 7.60 | 3.15 | 2.99 |
|  | 3.0 | 5.02 | 0.78 | 1.34 | 17.32 | 9.16 |  | 7.80 | 3.18 | 3.02 |
|  | 3.0 |  |  |  | 16.87 | 8.86 |  | 7.66 | 3.16 | 3.00 |
| Lace-wing | 4.0 | 6.08 | 0.88 | 1.65 | 24.63 | 9.42 |  | 7.25 | 4.11 | 3.94 |
|  | 4.0 | 5.33 | 0.13 | 1.43 | 25.71 | 9.91 |  | 7.85 | 4.18 | 4.02 |
|  | 4.0 | 3.99 | -1.21 | 1.04 | 25.22 | 9.61 |  | 7.66 | 4.16 | 4.00 |
|  | 4.0 |  |  |  | 25.30 | 9.87 |  | 7.48 | 4.14 | 3.97 |

TP*_k_* denotes the *known* trophic position of a given specimen; **^‡^** designates use of community-specific *β* mean, as opposed to the standard *β* (+8.4‰) used in terrestrial C_3_ plant food webs (Chikaraishi *et al.* 2011). Units for *δ*^15^N and Δ^15^N are ‰.

Δ^15^N_bulk_ = *δ*^15^N_consumer_ - *δ*^15^N_diet mean_;

TP_bulk_ = [(*δ*^15^N_consumer_ - *δ*^15^N_plant mean_)/3.4] + 1;

*β* = (*δ*^15^N_phe_ - *δ*^15^N_glu_)_basal resource_;

Δ^15^N_glu-phe_ = [(*δ*^15^N_consumer_ – *δ*^15^N_diet mean_)_glu_ – (*δ*^15^N_consumer_ – *δ*^15^N_diet mean_)_phe_;

TP_glu-phe_ = [(*δ*^15^N_glu_ – *δ*^15^N_phe_ + *β*_mean_)/7.6] + 1.

**Table S2.** Observations and calculations from the orchard food web.

| **Sample**  **type** | **TP_e_** | ***δ*^15^N_bulk_** | **Δ^15^N_bulk_** | **TP_bulk_** | ***δ*^15^N_glu_** | ***δ*^15^N_phe_** | ***β*** | **Δ^15^N _glu-phe_** | **TP_glu-phe_** | **TP^‡^_glu-phe_** |
| --- | --- | --- | --- | --- | --- | --- | --- | --- | --- | --- |
| Apple leaf | 1.0 | 2.65 |  | 1.03 | -2.07 | 7.43 | 9.50 |  | 0.86 | 1.05 |
|  | 1.0 | 2.49 |  | 0.98 | -6.38 | 3.72 | 10.1 |  | 0.78 | 0.97 |
|  | 1.0 | 2.55 |  | 1.00 | -7.74 | 2.19 | 9.93 |  | 0.80 | 0.99 |
|  | 1.0 | 2.52 |  | 0.99 | -5.19 | 4.68 | 9.87 |  | 0.81 | 1.00 |
| Aphids | 2.0 | 2.39 | -0.16 | 0.95 | 3.11 | 5.26 |  | 7.70 | 1.82 | 2.01 |
|  | 2.0 | 2.02 | -0.53 | 0.84 | 1.48 | 4.11 |  | 7.23 | 1.76 | 1.95 |
|  | 2.0 | 2.16 | -0.40 | 0.88 | 2.31 | 4.83 |  | 7.33 | 1.77 | 1.96 |
|  | 2.0 | 2.15 | -0.40 | 0.88 | -1.00 | 0.99 |  | 7.86 | 1.84 | 2.03 |
| Hover fly | 3.0 | 4.80 | 2.62 | 1.66 | 11.11 | 5.48 |  | 7.74 | 2.85 | 3.04 |
|  | 3.0 | 5.76 | 3.57 | 1.94 | 10.90 | 5.12 |  | 7.81 | 2.86 | 3.06 |
|  | 3.0 | 5.65 | 3.47 | 1.91 | 7.49 | 2.31 |  | 7.51 | 2.79 | 2.98 |
|  | 3.0 | 2.82 | 0.64 | 1.08 | 8.10 | 1.75 |  | 8.10 | 2.94 | 3.13 |
| Wasp  (1°) | 4.0 | 5.99 | 1.24 | 2.01 | 15.10 | 1.92 |  | 7.68 | 3.84 | 4.03 |
|  | 4.0 | 5.43 | 0.67 | 1.85 | 16.81 | 2.46 |  | 8.06 | 3.99 | 4.18 |
|  | 4.0 | 5.32 | 0.56 | 1.81 | 18.70 | 4.82 |  | 7.91 | 3.93 | 4.12 |
|  | 4.0 | 6.09 | 1.34 | 2.04 | 14.85 | 2.02 |  | 7.56 | 3.79 | 3.98 |
| Wasp  (2°) | 5.0 | 3.17 | -2.54 | 1.18 | 23.03 | 1.93 |  | 7.74 | 4.88 | 5.07 |
|  | 5.0 | 4.46 | -1.25 | 1.56 | 26.30 | 5.22 |  | 7.73 | 4.88 | 5.07 |
|  | 5.0 | 5.25 | -0.46 | 1.79 | 23.67 | 3.44 |  | 7.52 | 4.77 | 4.96 |
|  | 5.0 | 5.60 | -0.11 | 1.89 | 23.19 | 2.34 |  | 7.67 | 4.85 | 5.04 |

Units for *δ*^15^N and Δ^15^N are ‰. TP_e_ denotes the *expected* trophic position of a given specimen; **^‡^** designates use of community-specific *β* mean, as opposed to the standard *β* (+8.4‰) used in terrestrial C_3_ plant food webs (Chikaraishi *et al.* 2011). The primary (1°) parasitoid of the hover fly is distinguished from the secondary (2°) parasitoid (hyperparasitoid).

Δ^15^N_bulk_ = *δ*^15^N_consumer_ - *δ*^15^N_diet mean_;

TP_bulk_ = [(*δ*^15^N_consumer_ - *δ*^15^N_plant mean_)/3.4] + 1;

*β* = (*δ*^15^N_phe_ - *δ*^15^N_glu_)_basal resource_;

Δ^15^N_glu-phe_ = [(*δ*^15^N_consumer_ – *δ*^15^N_diet mean_)_glu_ – (*δ*^15^N_consumer_ – *δ*^15^N_diet mean_)_phe_;

TP_glu-phe_ = [(*δ*^15^N_glu_ – *δ*^15^N_phe_ + *β*_mean_)/7.6] + 1.
